# A Novel Phytotherapy Application: Preparation, Characterization, Antioxidant Activities and Determination of Anti-inflammatory Effects by *In vivo* HET-CAM Assay of Chitosan-based DDSs Containing Endemic *Helichrysum pamphylicum* P.H. Davis & Kupicha Methanolic Extract

**DOI:** 10.2174/1567201820666230328122504

**Published:** 2024-02-23

**Authors:** Nurlan Ismailovi, H. Tuba Kıyan, A. Alper Öztürk

**Affiliations:** 1 Department of Pharmaceutical Technology, Faculty of Pharmacy, Anadolu University, Graduate School of Health Sciences, Eskişehir, Türkiye;; 2 Department of Pharmacognosy, Faculty of Pharmacy, Anadolu University, Eskişehir, Türkiye;; 3 Department of Pharmaceutical Technology, Faculty of Pharmacy, Anadolu University, Eskişehir, Türkiye

**Keywords:** Antioxidant, chitosan gel, *Helichrysum pamphylicum* P.H. Davis & Kupicha, HET-CAM, polysaccharide, F9-HP

## Abstract

**Background:**

Numerous pharmaceutical applications for chitosan, a polysaccharide made from the shells of crustaceans by deacetylating chitin that occurs naturally, are currently being researched. Chitosan, a natural polymer, is successfully used to prepare many drug-carrier systems, such as gel, film, nanoparticle, and wound dressing.

**Objective:**

Preparing chitosan gels without external crosslinkers is less toxic and environmentally friendly.

**Methods:**

Chitosan-based gels containing *Helichrysum pamphylicum* P.H. Davis & Kupicha methanolic extract (HP) were produced successfully.

**Results:**

The F9-HP coded gel prepared with high molecular weight chitosan was chosen as the optimum formulation in terms of pH and rheological properties. The amount of HP was found to be 98.83% ± 0.19 in the F9-HP coded formulation. The HP release from the F9-HP coded formula was determined to be slower and 9 hours prolonged release compared to pure HP. It was determined that HP release from F9-HP coded formulation with the DDSolver program was by anomalous (non-fickian) diffusion mechanism. The F9-HP coded formulation significantly showed DPPH free radical scavenger, ABTS•+ cation decolorizing and metal chelating antioxidant activity while weakly reducing antioxidant potential. According to the HET-CAM scores, strong anti-inflammatory activity was obtained by the F9-HP coded gel at a dose of 20 µg.embryo^-1^ (*p*<0.05 compared with SDS).

**Conclusion:**

In conclusion, it can be said that chitosan-based gels containing HP, which can be used in both antioxidant and anti-inflammatory treatment, were successfully formulated and characterized.

## 1 INTRODUCTION

An essential component of contemporary medicine and pharmacy are phytopharmaceuticals, prepared and derived from medicinal plants containing a wide range of bioactive molecules, including phenolic compounds, simple phenolics, phenolic acids, anthocyanins, hydroxycinnamic acid derivatives, and flavonoids [1, 2]. These effective chemical groups have been used to treat various disorders and diseases, including hypertension, obesity, cancer, diabetes, atherosclerosis, and many other conditions. Recently these bioactive phenolic classes have drawn much attention due to their physiological properties, including free radical scavenging and antioxidants. The primary cause of phenolics' redox characteristics, which enable them to function as reducing agents, hydrogen donors, and singlet oxygen quenchers, compose their antioxidant activity. They may also chelate metals [2, 3].

*Helichrysum* (Asteraceae) plants are aromatic plants referred to as everlasting, immortal and fadeless flowers and are utilized extensively in traditional medicine worldwide. The potential of these plants for drug discovery has been discovered through investigations on their traditional usage as well as phytochemical and pharmacological ones. This genus, which has approximately 600 species and is extensively distributed throughout the world, especially widespread through Eurasia, Africa, and Australia, is represented by 27 taxa in the Turkish flora belonging to 21 species, 15 of which are endemic and are common in Anatolia [4-8]. A review of studies revealed that *Helichrysum* species had been used in various traditional and folk medical systems to treat various infections, wounds, digestive issues, diabetes, and colds, some of which have been verified in contemporary medicine, such as the antibacterial activity. They have also been used for millennia for aesthetic purposes and flavoring spices in various dishes and traditional treatments [4]. Due to these plants' flavonoids, their aerial parts have been utilized as daily herbal tea for their therapeutic benefits. They have been used to treat gall bladder diseases in folk medicine for at least 2000 years because of their bile control, anti-infective, hepatoprotective, detoxifying, choleretic, and diuretic activities. They are also used as wound dressings and to treat wounds, coughs, and erythema [8].

*Helichrysum* phenolic compounds have biological effects like anti-inflammatory, immune-stimulating, antiallergenic, anti-atherogenic, and antibacterial properties. They are also used to treat disorders like coronary heart disease, stroke, and cancer. Their essential oils have anti-inflammatory, antiviral, antifungal, and antioxidant activities [6, 9-11]. Under the names “altınotu” or “ölmez çiçek,” *Helichrysum* species are often consumed in Turkey as herbal teas to treat many disorders [2]. Additionally, they are frequently used for their many biological properties, such as their anti-inflammatory, antioxidant, diuretic, and antimicrobial activities, removal of kidney stones, and treatment of urogenital disorders, jaundice, diarrhea, and asthma [12, 13].

There are very few records of the endemic *Helichrysum pamphylicum* P.H. Davis & Kupicha of the Turkish flora, even though the biological activities of several *Helichrysum* species have previously been researched [14].

Excellent mechanical and physical characteristics of chitosan allow it to be transformed into various dosage forms, including film, gel, nanoparticles, microparticles, and nanofibers, as well as to accept a wide range of processing techniques [15, 16]. Chitosan is one of the most intriguing biopolymers in this field because of its potential qualities for biomedical engineering applications, such as biodegradability, biocompatibility, and non-toxicity. Because of this, chitosan and its derivatives have garnered considerable interest in a wide range of biomedical applications [17]. The myriad biological and technological traits of chitin and chitosan have sparked interest in these polymers [18]. These can be listed as follows: Anti-inflammatory [19, 20], antifungal [21], antihyperglycemic [22], antimicrobial [23], antioxidant [19], antitumoral [24], mucoadhesive [25], and wound healing [26].

The term “gel” is typically used to refer to highly hydrated networks that contain two components in varying amounts: the solvent, which dominates in mass, and the polymeric solute, which are typically either natural or synthetic macromolecules. The macromolecule used must be able to effectively retain a significant amount of solvent [27]. Gel usage is ubiquitous and expanding, particularly in the biomedical industry [28]. Preparing chitosan gels without external crosslinkers is less toxic and environmentally friendly and is a very important approach in pharmaceutical technology and biomedicine [29].

To the best of our knowledge, no previous research on the *in vitro* antioxidant and *in vivo* anti-inflammatory activity of *Helichrysum pamphylicum* P.H.Davis & Kupicha methanolic extract (HP) loaded chitosan gel using the HET-CAM assay has been published.

## 2 MATERIALS AND METHODS

### 2.1 Materials

ABTS^●+^, BHT, DPPH•, EDTA, ethanol, FeCl_2_, FeCl_3_, Ferrozine, K_2_S_2_O_8_, K_4_ [Fe (CN)_6_].3H_2_O, Lactic acid, Methanol, potassium phosphate dibasic, potassium phosphate monobasic, Sodium bicarbonate, Sodium hydroxide, sodium salicylate, TCA and Vit C were purchased from Sigma-Aldrich, Germany. Agarose was purchased from Fluka, Spain. Chitosans of different molecular weights (HMW, LMW and MMW) were purchased from Sigma-Aldrich, Iceland. SDS was purchased from Fluka-Biochemika, Germany. Also, the fertilized eggs used in the HET-CAM test were obtained from Has Tavuk Company (Bursa and Sivrihisar, Turkey). All other chemicals used were of analytical grade.

### 2.2 Plant Material

The endemic *Helichrysum pamphylicum* P.H. Davis & Kupicha was collected from Gazipaşa, Antalya, in June 2021.

### 2.3 Preparation of Plant Extraction

Dried aerial parts of the plant at room temperature were ground to a fine powder with a grinder. Then the powdered plant material (25 g) was extracted using an ultrasonic bath with 100 mL methanol (MeOH) at room temperature (25°C) for 2 h, at least three times. Thereafter, the extracts were combined, filtered, and evaporated to dryness under a vacuum at 40°C with a rotary evaporator. After determining the yield, the extract was dissolved in methanol or DMSO for further study.

### 2.4 Preparation of Formulations

Chitosan-based blank gel formulations were prepared with minor modifications after a literature review [30-34]. Blank gel formulations were prepared by dissolving chitosan polymer at different rates and different molecular weights in 1% (v/v) aqueous lactic acid. Methyl sodium salicylate (0.2%, w/w) was added to the tested samples as a preservative. The samples were mixed on a magnetic stirrer at 250 rpm, at room temperature, and the resulting blank gels were sonicated to remove air bubbles. The air bubbles removed gels were taken into sample containers for analysis, and pH measurements were carried out.

Chitosan gel formulations containing HP methanol extract were prepared to contain 0.1% (10 mg in 10 grams of gel) of HP methanol extract. As in the blank gel formulations, the samples were mixed on a magnetic stirrer at 250 rpm, at room temperature, and the resulting gels were sonicated to remove air bubbles. The air bubbles removed gels were taken into sample containers for analysis, and pH measurements were carried out. First, pH was measured in chitosan gels containing blank and HP methanol extract. The formulations were prepared again using sodium bicarbonate for pH adjustment, and pH measurements were performed. e contents of the formulations prepared within the scope of this study are presented in Table **1**.

### 2.5 Characterization of Formulations

#### 2.5.1 Evaluation of the Physical Appearance of Formulations

Clarity is one of the key characteristics of the gels. The clarity of all prepared formulations was checked visually against a white background. Visual inspection also assessed other physicochemical qualities like appearance, transparency, and color. The homogeneity of all formulations was evaluated by visual and tactile examination of the prepared gel samples after they were well placed in the white-capped sample cups. The gels were examined for the presence of any aggregates, their appearance, and the type of stain. Images were taken to support these analyses.

#### 2.5.2 Determination of pH Values of Formulations

Because of the three zones of critical importance mentioned below, pH is the most important factor: the effects of pH on durability, solubility, and epidermis. Any gel should also have a pH that does not irritate the patient and ensures the durability of the formulation. The pH values of formulations were determined using a digital pH meter (Mettler Toledo^TM^ S220 Seven Compact^TM^ pH / lon Benchtop Meter). Measurements were repeated 3 times [35]. Technical characteristics of the device used: Parameter: pH; Ion; ORP, Channel: Single channel, pH measuring range: -2 – 20, pH solubility: 0.001; 0.01; 0.1, pH accuracy (±): 0.002, Ion concentration measurement range: 1.00E-9 – 9.99E+9, Ion concentration accuracy (±): 0.5%, Temperature range: -30°C - 130°C, Temperature resolution: 0.1°C, Temperature accuracy (±): 0.1°C.

#### 2.5.3 Determination of the Rheological Behavior of Formulations

Rheological properties were determined using a 40 mm diameter cone-plate geometry rheometer (Brookfield, USA). Measurements and viscosity changes were repeated three times at 25 ± 1°C. Shear stress *versus* shear velocity graphs were created, and compliance with the flow models was evaluated [36].

#### 2.5.4 Analytical Validation Study for Helichrysum Pamphylicum Methanol Extract

A stock solution was prepared to determine the maximum absorbance (λ_max_) of the HP. Exactly 10 mg of HP was prepared by washing it with pH 5.5 PBS, taking it into a flask, adding 10 mL volume to pH 5.5 PBS and keeping it in an ultrasonic bath for ten minutes. Based on the concentration of the resulting stock solution being 1000 µg.mL^-1^, a solution was prepared at a concentration of 100 µg.mL^-1^ by dilution. The resulting solution was scanned in the UV range (200-400 nm) [37]. The average of three original curves generated with samples of HP solution at seven concentration levels ranging from 5.0 to 60.0 µg.mL^-1^ was used to evaluate the linearity of the method. Solutions were prepared by diluting the stock standard solution (1000 µg.mL^-1^) in pH 5.5 PBS. Absorbances were measured in triplicate at 308 nm. The curve was constructed by representing the mean values of absorbance *versus* concentration. The results were statistically analyzed by linear regression using the least squares method [38]. In the precision studies of the analytical method belonging to HP, in order to show its reproducibility, 3 different concentrations (25 µg.mL^-1^, 35 µg.mL^-1^, 45 µg.mL-1) correspond to the calibration range. HP-containing solutions were prepared, and 3 measurements were repeated for each concentration. Recovery was determined by adding known increasing amounts of standard HP (25 µg.mL^-1^, 35 µg.mL^-1^, 45 µg.mL^-1^) solution to the samples at 100% of the concentration analysis. Recovery values are expressed as percentages for the experimentally determined total HP ratio and theoretical concentrations. Each sample was tested three times, and the amount recovered was calculated. The specificity/selectivity of the method was determined by looking at the overlap in the spectra of the samples obtained in the 200-800 nm range of the standard solution (HP), pH 5.5 PBS and blank formulation (F9) [39, 40].

#### 2.5.5 Determination of Helichrysum Pamphylicum Methanol Extract Amount in Formulations

25 mL of pH 5.5 PBS was used to dissolve 0.5 g of precisely weighed gel. The reaction flask containing the gel solution was agitated for two hours on a magnetic stirrer to obtain the total solubility of the HP. This solution was filtered using a membrane filter (0.45 μm), and following the necessary dilutions, it was examined at 308 nm.

#### 2.5.6 *In vitro* Dissolution/Release Study

The *in vitro* dissolution studies of formulation were performed using the dialysis bag diffusion technique equipped with a magnetic stirrer (IKA^®^ Labortechnik RT 15 S000, Germany) at a speed of 100 rpm. Briefly, 2.5 mg of HP and gel containing HP equivalent to 2.5 mg HP was suspended in 1 mL of pH 5.5 PBS and transferred into a dialysis bag (Dialysis tubing cellulose membrane average flat width 33 mm (1.3 in.), MWCO: 14.000, D9652, Sigma-Aldrich, USA). The dialysis bags were placed into a beaker containing 80 mL of pH 5.5 PBS at 37°C±1°C. The receptor compartment/beaker was closed to prevent evaporation of the release medium. Samples of the medium (3 mL) were withdrawn and replaced with fresh medium at 1, 2, 3, 4, 5, 6, 9, 12 and 24 hours. HP concentration in the samples was quantified by UV-spectrophotometer (308 nm). The *in vitro* dissolution study was repeated three times for F9-HP and pure HP, then the results were calculated as Mean ± SD. The results were then plotted as the cumulative release [41].

#### 2.5.7 Release Kinetics

Data from the *in vitro* drug release studies were investigated for release kinetics using the DDSolver software program [42-44].

### 2.6 Antioxidant Efficacy Studies

#### 2.6.1 *In vitro* Metal Chelating Effect

The samples' ability to chelate ferrous ions was calculated using a modified version of the approach [45]. 40 µL of a 2 mM FeCl_2_ solution were incubated with 1480 µL of methanol and 400 µL of the samples. 80 µL of 5 mM ferrozine was added to the mixture to start the reaction, which was then allowed to proceed for 15 min at room temperature. At 562 nm, the reaction mixture's absorbance was gauged. EDTA was used as a positive control of the assay. The following formula was used to determine the ratio of inhibition of ferrozine-Fe^2+^ complex formation (Eq. 1.):


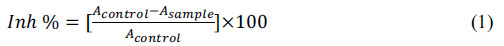


#### 2.6.2 *In vitro* ABTS●+ Radical Cation Decolorization Assay

The samples' ABTS+ radical cation decolorization effect was calculated using a modified version [46]. By combining ABTS stock solution (7 mM) with 2.45 mM potassium persulfate (final concentration) and letting the combination sit undisturbed at room temperature for 12 to 16 hours before use, ABTS radical cation (ABTS•1) was created. The ABTS^•1^ solution was diluted with ethanol to an absorbance of (0.70 ± 0.02) at 734 nm and equilibrated at room temperature to examine the samples. After being added to 10 µL of positive standards such as ascorbic acid, BHT, and samples in ethanol or DMSO in water 50%, 1.0 mL of diluted ABTS^•1^ solution (A_734nm_=0.700 ± 0.020) was added. The mixture was then allowed to remain at room temperature for 5 minutes. At 734 nm, the reaction mixture's absorbance was gauged. Following is how the ratio of decolorization was determined (Eq. 2):


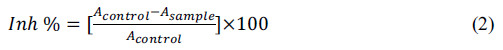


#### 2.6.3 Reducing Power Activity

The improved method of determining the reducing power of samples was used [47]. Samples were combined with 500 µL of phosphate buffer (200 mM, pH 6.6) and 500 µL of 1% potassium ferricyanide in varying concentrations. At 50°C, the mixtures were incubated for 20 min. 500 µL of 10% trichloroacetic acid was added to the mixtures after incubation, and the mixtures were then centrifuged at 4000 rpm for 10 minutes. The absorbance of the resulting solution was determined at 700 nm after mixing the upper layer (500 µL) with 200 µL of 0.1% ferric chloride and 500 µL of distilled water. Greater reducing power resulted from increased reaction absorbance. The term “EC_50_” refers to the concentration that produces an absorbance of 0.5 at 700 nm. Therefore, a lower EC_50_ suggested a higher reducing power.

#### 2.6.4 *In vitro* Free Radical Scavenging Assay (DPPH test)

The scavenging effects of the samples on DPPH free radicals were determined using a modified method of Sánchez‐Moreno [48]. The free radical scavenging activities of the samples were expressed as a percentage of inhibition calculated according to Eq. (3). In Eq. (3), A_control_ is the absorbance of the control (containing all reagents except the test compound), A_sample_ is the absorbance of the sample with added DPPH. The IC_50_ values were obtained by plotting the DPPH scavenging percentage of the sample against the sample concentration. All the data determined from the *in vitro* antioxidant assays were analyzed using the SigmaPlot software (Version 14.5), and the IC_50_ values were obtained (Eq. 3).


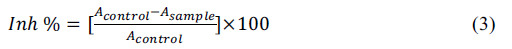


### 2.7 *In vivo* Anti-inflammatory Activity / HET-CAM Assay

#### 2.7.1 Preparation of Test Samples

Positive standard sodium dodecyl sulfate (SDS, 2 mg.mL^-1^), negative standard hydrocortisone (2 mg.mL^-1^) were dissolved in 1 mL 2.5% (w/v) agarose solution, and HP (1 and 2 mg.mL^-1^) was suspended in 1 mL 2.5% (w/v) agarose solution with SDS (1 and 2 mg.mL^-1^). For ease of application, the pellets of these solutions (10 µL) were prepared and applied dropwise on circular stainless-steel supports of 5 mm diameter and cooled to room temperature for solidification and applied onto the chick chorioallantoic membrane (CAM) [49, 50]. Also, 10 µL and 20 µL of the gel formulation (1 mg.mL^-1^) and blank with SDS (2 mg.mL^-1^) were applied on the CAM, which had approximately a diameter of 2 cm.

#### 2.7.2 *In vivo* HET-CAM Assay

Determining the anti-inflammatory properties of herbal materials [51, 52]. Compared to comparable *in vivo* trials, the HET-CAM test, an alternative *in vivo* animal assay to the Draize rabbit eye test, is less unethical [53, 54]. The HET-CAM test was carried out per our prior study [50, 55]. The previously fertilized hen's eggs were incubated for 72 hours at 36.5°C and 80% relative humidity by being put horizontally and rotating a few times. The eggs were cracked open on the snub side, and another 10-15 mL of albumin was removed. After tracing the eggs' shells with a scalpel at the height of two-thirds, the eggs' shells were then removed using forceps (from the pointed side). The cavity was covered with film and incubated for 72 hours at 36.5°C with a relative humidity of 80%. Test treatments were applied to the CAMs of the eggs on day 6. One additional day later, the eggs were seen under a stereo microscope. A total of 10 to 15 eggs were utilized for each test chemical.

The effectiveness of the anti-inflammatory effects was evaluated using a scoring method, and the proportionate inhibition of inflammation was then calculated from the score index (Tables **2** and **3**). A typical SDS-induced heavily vascularized granuloma with star-like capillaries around the pellet or gel formulation was seen on the CAM. When SDS and anti-inflammatory test chemicals are mixed, membrane irritation returns to normal.

### 2.8 Statistical and Mathematical Analysis

We used DDSolver and Microsoft Excel for our mathematical investigation. GraphPad Prism version 7.0 performed a one-way ANOVA test on all the collected data. At least three replicates of each measurement were performed. P 0.05 was regarded as statistically significant in each study. Each of the results was displayed as Mean±SD [56-58].

## 3 RESULTS AND DISCUSSION

### 3.1 Preparation of Formulations

This work determined the total extract yield, antioxidant activity, and gel formulation, including HP. The yield of the methanolic extract was 4.00%.

Because of chitosan's ability to function in many forms, it has many areas of interest in the medical industry, including orthopedic and periodontal applications, tissue engineering, wound healing, and drug delivery systems. Chitosan is very popular in the biomedical field due to its healing accelerator, hemostatic agent, antibacterial agent, antifungal agent, weight loss aid effects, artificial skin, surgical sutures, artificial blood vessels, controlled drug release, contact lenses, eye washes, bandages, sponges, burn dressings, blood cholesterol control, anti-inflammatory effect, tumor inhibition, antiviral effect, plaque inhibition and bone healing treatment [59]. Chitosan gel formulations containing different active ingredients designed for different purposes are available in the literature [30-34]. Chitosan was preferred in this study because of its many important properties.

### 3.2 Evaluation of the Physical Appearance of Formulations

The images of the prepared and optimized gels are presented in Fig. (**1**). While pure, smooth, clear, and slightly yellow color due to chitosan was obtained in blank gels, a more yellowish image was obtained in chitosan gels containing HP due to HP. The smoothness of the prepared gels containing HP showed their suitability for topical use. After the visual and flow macroscopic examination of the gels containing HP, different properties were noticed. The best viscosity and flow properties were observed in F9 (blank), and F9-HP coded formulations prepared with a 1.5% (w/v) concentration of chitosan (HMW).

### 3.3 Determination of pH Values of Formulations

**Fig. (2):**
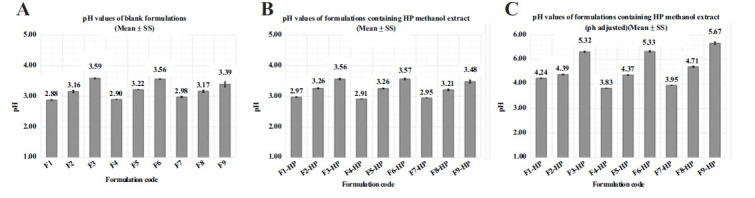
pH analysis results of formulations. (**A**): pH results of blank gels without pH adjustment, (**B**): pH results of gels containing HP without pH adjustment, (**C**): pH results of gels containing HP with pH adjustment.

The normal pH of the body skin surface (Stratum corneum-SC) is acidic. Normal skin has a pH in the range of 4.1-5.8 PSmall pH differences can be observed between the face, trunk, and extremities [60, 61]. The acidic pH of the skin surface is recognized as a regulatory factor for maintaining SC homeostasis and barrier permeability. There is a high level of agreement that topical products are suitable for normal skin pH and should have a pH between 4 and 6 [62, 63]. When the literature is searched, it is seen that pH adjustment is made for the preparation of formulations in the normal skin pH range, or the pH values of the prepared formulations with normal skin pH range are optimally selected [64-66]. The pH values of the blank gels coded F1, F2, F3, F4, F5, F6, F7, F8 and F9 were obtained as 2.88 ± 0.01, 3.16 ± 0.03, 3.59 ± 0.01, 2.90 ± 0.00, 3.22 ± 0.00, 3.56 ± 0.01, 2.98 ± 0.02, 3.17 ± 0.04 and 3.39 ± 0.08, respectively (Fig. **2A**). The pH values of the HP containing gels coded F1-HP, F2-HP, F3-HP, F4-HP, F5-HP, F6-HP, F7-HP, F8-HP, and F9-HP were obtained as 2.97 ± 0.01, 3.26 ± 0.02, 3.56 ± 0.02, 2.91 ± 0.00, 3.26 ± 0.01, 3.57 ± 0.03, 2.95 ± 0.00, 3.21 ± 0.03 and 3.48 ± 0.05, respectively (Fig. **2B**). The obtained pH values may irritate the skin structure, and adjustment is thought necessary. The pH values of the gels coded F1-HP, F2-HP, F3-HP, F4-HP, F5-HP, F6-HP, F7-HP, F8-HP and F9-HP, whose pH was adjusted with sodium bicarbonate, were obtained as .24 ± 0.10, 4.39 ± 0.00, 5.52 ± 0.01, 3.38 ± 0.01, 4.37 ± 0.01, 5.33 ± 0.02, 3.95 ± 0.01, 4.71 ± 0.03 and 5.67 ± 0.02, respectively (Fig. **2C**). F9-HP, the formulation with the best viscosity and flow properties after macroscopic and physical examinations, was chosen as optimum since the pH value was considered, and the remaining studies were carried out on F9-HP [62, 63].

### 3.4 Determination of the Rheological Behavior of Formulations

**Fig. (3):**
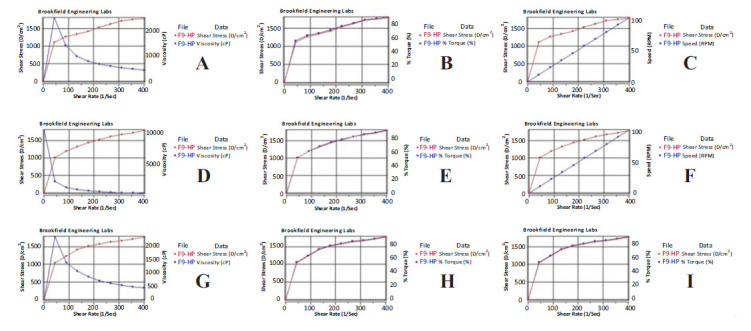
Rheograms obtained in rheological examinations for F9-HP coded gel. (**A**, **B**, **C**): Results for measurement 1; (**D**, **E**, **F**): Results for measurement 2; (**G**, **H**, **I**): Results for measurement 3.

In the rheological examinations, it was observed that the viscosity decreased as the shear stress increased for the F9-HP coded gel (Fig. **3**). In a previous gel formulation study, rheograms were shown showing an inverse relationship between shear stress and viscosity. In the related study, the increase in shear stress decreased the viscosity. The rheological examination of the gels prepared in the relevant study stated that due to viscosity reduction, non-Newtonian pseudoplastic flow behavior was observed [67]. It can be said that the F9-HP-coded gel showed pseudoplastic flow.

**Fig. (4):**
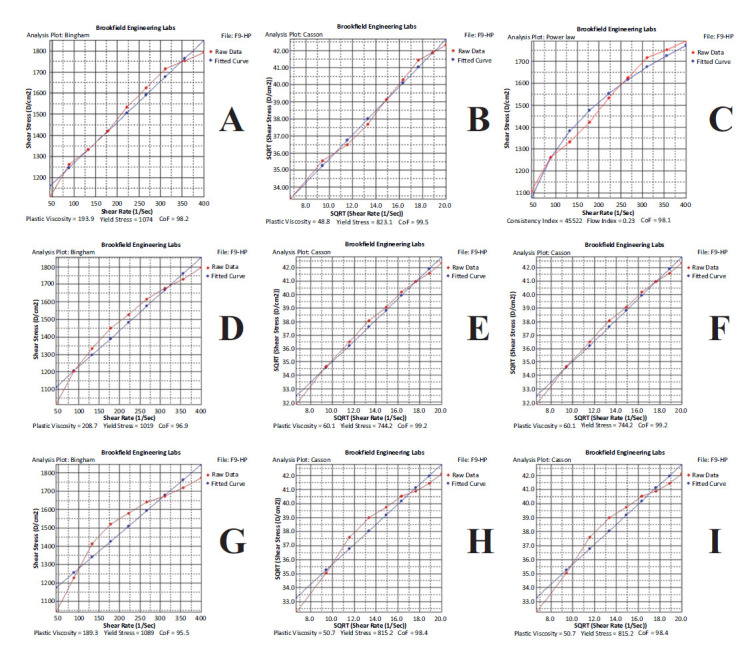
Flow models obtained in rheological examinations for the F9-HP coded gel. (**A**, **B**, **C**): Results for measurement 1; (**D**, **E**, **F**): Results for measurement 2; (**G**, **H**, **I**): Results for measurement 3.

After determining the flow type of the F9-HP coded formulation, it was examined which flow model it was suitable for. For this purpose, different rheological flow models based on shear stress-shear rate data were tested using the software provided with the rheometer. When the coefficients of friction (CoF) values are examined in the model analysis, the average of the three measurements is obtained as 96.867% ± 1.350, 99.033% ± 0.569, and 98.700% ± 0.794 for Bingham, Casson and Power Law models, respectively (Fig. **4**). As can be seen from the mathematical results and Fig. (**4**), higher CoF values were obtained in Casson and Power Law models, and a high correlation was found between these two models. Considering all the rheological analyzes and considering that the prepared formulation is a system showing pseudoplastic flow, it can be said that the most appropriate flow model is Power Law. Bingham and Casson's models describe plastic systems, while the Power-law model best suits pseudoplastic (shear thinning) systems. It can be said that the most suitable model for the F9-HP coded gel formulation is the Power Law model, which shows that this formula does not show a visible yield value and shows limited resistance to flow at low-stress values, which is characteristic of pseudoplastic flow. This shear-thinning behavior is desirable for topical preparations as they must be thin at the time of application and otherwise thick/solid [68].

### 3.5 Determination of HP Amount in Formulations

When the literature is examined, the UV-spectrophotometry method has been successfully used in quantitative analyzes of formulations containing avocado peel extract [69], *Pterocarpus marsupium Roxburgh* extract [70], *Agrimonia eupatoria L.* extract [71], *Clitoria Ternatea* petal extract [72], cardamom extract [73], pomegranate extract [74] and ginger extract [75]. UV-spectrophotometry method was used because of its simplicity, speed, sensitivity, equipment availability and relatively low reagent costs [76, 77].

**Fig. (5):**
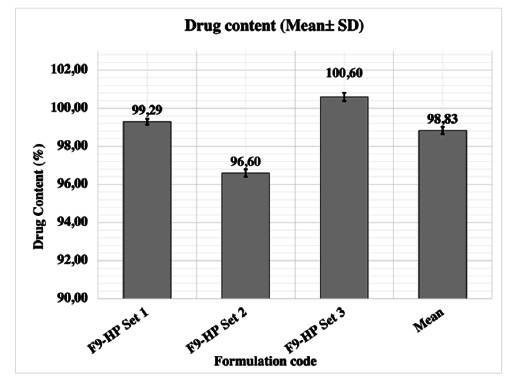
Drug content results.

Following validation of the developed UV–Visible spectrophotometric method, linearity was determined to be at a concentration range of 5.0–60.0 μg.mL^−1^ with linearity of y = 0.0099x + 0.0011 (r^2^ = 0.9999). The method was decided to be precise due to relative standard deviation (RSD) values of <2% for repeatability and intermediate precision. Recovery and accuracy of the method were satisfactory owing to < 2% RSD value. Accuracy values of 99.392% ± 0.608, 99.629% ± 0.371 and 99.686% ± 0.314 for concentrations of 25, 35 and 40 μg.mL^−1^, respectively, were determined for the UV–Visible spectrophotometric method. In the selectivity study, blank formulations (F9) and pH 5.5 PBS were photometrically examined between 200 and 800 nm and did not yield any absorbance peak at 308 nm. Consequently, the easy and inexpensive procedure proposed in this study can be used for routine and simultaneous HP determination [78]. As a result of quantification, it was concluded that 98.83% ± 0.19 of HP was loaded on gels (Fig. **5**).

### 3.6 *In vitro* Dissolution / Release Study

*In vitro* release profile of pure HP and F9-HP coded formulation are shown in Fig. (**6**). HP showed a release rate of 79.838% ± 3.095 at the end of the first hour and 97.397% ± 2.086 at the end of the second hour in pH 5.5 PBS medium. At the end of the 3^rd^ hour, HP was released in pH 5.5 PBS medium with a release rate of 100.348% ± 1.373. Considering the HP release rates from the F9-HP coded chitosan gel formulation containing HP, an HP release rate of 55.278% ± 5.385 was observed at the end of the 3^rd^ hour. The HP release from the F9-HP coded chitosan gel formulation was 99.867% ± 1.969 at the end of the 9^th^ hour, and the HP was released from the F9-HP coded chitosan gel formulation. According to the literature, it is quite clear that the HP release from the F9-HP formula is slower and 9 hours prolonged than the pure HP [79-81].

### 3.7 Release Kinetics

After obtaining the release profiles, data were transferred to the DDSolver program to determine the four most important and popular criteria: coefficient of determination (Rsqr, R^2^, or COD), adjusted coefficient of determination (Rsqr_adj or R^2^_adjusted_), Akaike Information Criterion (AIC), Model Selection Criterion (MSC). The highest R^2^, R^2^_adjusted_ and MSC values and the lowest AIC values were used for evaluating Zero-order, Zero-order (T_lag_), Zero-order (F_0_), First-order, First-order (T_lag_), First-order (F_max_), First-order (T_lag_ and F_max_), Higuchi, Higuchi (T_lag_), Higuchi (F_0_), Korsmeyer-Peppas, Korsmeyer-Peppas (T_lag_), Korsmeyer-Peppas (F_0_), Hixson-Crowell, Hixson-Crowell (T_lag_), Hopfenberg, Hopfenberg (T_lag_), Baker-Lonsdale, Baker-Lonsdale (T_lag_), Peppas-Sahlin, Peppas-Sahlin 1 (T_lag_), Peppas-Sahlin 2, Peppas-Sahlin 2 (T_lag_), Quadratic, Quadratic (T_lag_), Weibull 1, Weibull 2, Weibull 3, Weibull 4, Logistic 1, Logistic 2, Logistic 3, Gompertz 1, Gompertz 2, Gompertz 3, Gompertz 4, Probit 1, and Probit 2 models [42, 82].

When Table **4** was examined, a high correlation was obtained between Korsmeyer-Peppas and Peppas-Sahlin models in the first 6 hours for the F9-HP coded formulation. This correlation indicates that the systems prepared with high molecular weight chitosan do not have a single release mechanism but release with more than one mechanism [42]. The literature has previously reported that releasing the active substance from drug delivery systems can fit more than one model [83].

Since a high correlation was observed between the models, especially in the Korsmeyer-Peppas model, the '*n*' value is the diffusion exponent indicating the drug release mechanism. The *n* value related to the release mechanism can have different values and ranges. These values and ranges can be as follows; *n* < 0.5, *n* = 0.5, 0.5 < *n* < 1.0, *n* = 1 or *n* > 1.0. If *n* < 0.5, the drug delivery system releases by the semi-fickian diffusion mechanism; if *n*=0.5, the drug delivery system releases by the fickian diffusion mechanism; if 0.5 < *n* < 1.0, the drug delivery system releases by anomalous (non-fickian) diffusion mechanism It has been reported in the literature that if *n*=1, the drug delivery system releases by non-Fickian state II mechanism, and if *n* > 1.0, the drug delivery system releases by non-Fickian superstate II mechanism [84]. For the Korsmeyer-Peppas model of the F9-HP coded formulation, the *n* value was observed as 0.619 in the first 6 hours of release kinetics. In line with this information, it can be said that the drug delivery system prepared in this study releases by anomalous (non-fickian) diffusion mechanism.

### 3.8 Antioxidant Efficacy Studies

Free radicals and reactive oxygen species (ROS), which occur under typical physiological settings but turn harmful when not removed by endogenous processes, are the results of oxidative stress [85]. Oxidative stress is brought on by an imbalance between endogenous antioxidant mechanisms and the production of reactive oxygen species [86, 87]. Numerous diseases and disorders, including cancer, cardiovascular disease, obstructive pulmonary disease (COPD), and neural disorders such as Alzheimer's disease, mild Parkinson’s disease, alcohol-induced liver disease, ulcerative colitis, aging, and atherosclerosis, are caused by oxidative stress [85-90].

Degenerative illnesses are more likely to develop when antioxidants, which can neutralize these reactive free radicals, are deficient in the body. Because of their antioxidant activity, phenolic compounds, abundant in plant extracts, have various biological effects, including anti-inflammatory, anti-carcinogenic, and anti-atherosclerotic [91]. Thus, since the overproduction of oxidants (reactive oxygen species and reactive nitrogen species) in the human body is involved in the pathogenesis of many chronic diseases, the protective role of these phytochemicals, including tannins, flavones, triterpenoids, steroids, saponins, and alkaloids, may be associated with their antioxidant activity [92].

#### 3.8.1 *In vitro* Metal Chelating Effect

The ferrous ion-chelating assay was used to determine the metal-chelating activity of the samples, and the results are shown in Table **5**. Compared to EDTA, F9-HP had the greatest effect (IC_50_=0.3790 mg.mL^-1^), while HP had the least chelating potential. Chitosan also demonstrated antioxidant potential, and the antioxidant effects of the samples were identified as EDTA ˃ F9-HP ˃ F9 (Blank) ˃ HP.

#### 3.8.2 *In vitro* Antioxidant-ABTS Radical Decolorization Activity

As shown in Table **6**, when *in vitro* ABTS radical decolorization activity of samples was evaluated, F9-HP had the highest antioxidant potential (IC_50_=0.5120 mg.mL^-1^), while HP had the lowest when compared to BHT and ascorbic acid. The antioxidant potentials ranged from ascorbic acid to BHT to F9-HP to HP.

#### 3.8.3 *In vitro* Reducing Power Activity

When reducing power results were compared to positive standards, HP had the highest antioxidant potential (EC_50_=1.8587 mg.mL^-1^), while F9-HP had the lowest. The antioxidant effect of F9-blank was not observed. Table **7** shows the antioxidant activity range for ascorbic acid, BHT, HP, and F9-HP.

#### 3.8.4 *In vitro* DPPH Radical Scavenging Activity

The samples' capacity to scavenge free radicals was assessed using the DPPH technique, and the findings are shown in Table **8**. DPPH is a valuable tool for analyzing a compound's capacity to scavenge free radicals. The extracts successfully reduced the stable radical DPPH in the DPPH test to the yellow-colored diphenylpricrylhydrazine. The process relies on reducing an alcoholic DPPH solution in the presence of an antioxidant that donates hydrogen because the reaction produces the non-radical form of DPPH-H [2].

The HP methanolic extract exhibited the highest antioxidant capability (EC_50_=0.0023 mg.mL^-1^), while F9-HP had the lowest when DPPH scavenging activity data were compared to BHT. F9-blank did not have an antioxidant effect. The range of antioxidant activity for BHT, HP, and F9-HP is displayed in Table **8**.

Chitosan, which has been previously documented to have antioxidant capabilities, and HP appear to display antioxidant potential synergistically in the gel formulation when *in vitro* antioxidant activity results are reviewed together [93].

Despite numerous papers addressing the antioxidant activity of various *Helichrysum* species, there is insufficient information regarding the antioxidant and free radical scavenging properties of the Turkish flora's endemic *Helichrysum pamphylicum.* Antioxidant and antiradical activities of the methanolic extract from *H. pamphylicum* collected from different regions of Turkey have been reported in a previous study. In this paper, the antioxidant activity of HP was reported to show DPPH radical scavenging activity with an IC_50_ value of 15.21 µg.mL^-1^ which is significantly lower than our antioxidant activity result (IC_50_=2.3 µg.mL^-1^) [14].

### 3.9 The *In vivo* Anti-inflammatory Activity / HET-CAM Assay

In a physiologically healthy body, angiogenesis, also known as neovascularization, plays a critical role in tissue repair, wound healing, embryogenesis, and the development of the female reproductive system [94]. Pathological manifestations of pathologies include cancer, persistent inflammation, cardiovascular disease, autoimmune disorders, diabetic retinopathy, psoriasis, endometriosis, and obesity-related angiogenesis [95]. Neovascularization may be seen in inflamed lesions and be one of the histological findings of many inflammatory disorders, including rheumatoid arthritis, according to research on the relationship between angiogenesis and inflammation. As a result, the relationship between angiogenesis and inflammation is viewed favorably [20].

*In vivo* Hen's Egg Test on the Chorioallantoic Membrane (HET- CAM) assay is well established to screen anti-inflammatory, teratogenic, potentially toxic and side effects and irritation potency of natural products, including plant extracts and essential oils as well as synthetic therapeutics, drug formulations, and cosmetics. It is also very useful, affordable, and simple to perform [20, 95]. The anti-inflammatory activity of samples was tested *in vivo* using the HET-CAM assay. SDS was used in the assay to cause irritation and hemorrhage on the CAM. Table **9** summarizes the anti-inflammatory results. As shown in Table **2**, a semi-quantitative score system and a score index, as described in Table **3**, were used to assess the anti-inflammatory effect [20].

**Fig. (7):**
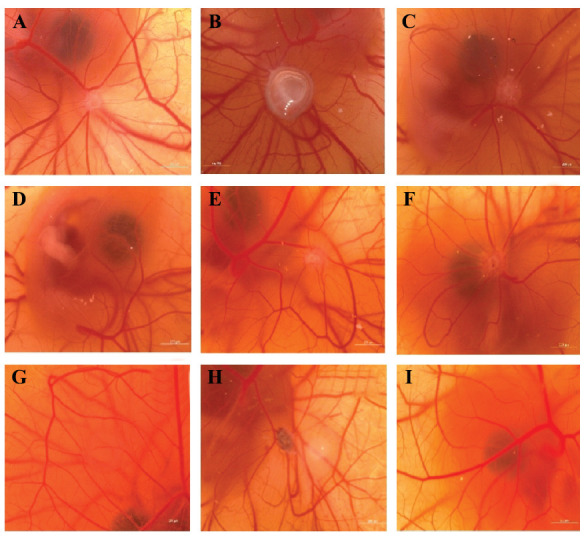
The *in vivo* anti-inflammatory effects of samples on the CAM. (**A**): HP (10 µg.pellet^-1^, uncertain anti-inflammatory effect), (**B**): HP (20 µg.pellet^-1^, weak anti-inflammatory effect), (**C**): F9-HP (10 µL.embryo^-1^, weak anti-inflammatory effect), (**D**): F9-HP (20 µL.embryo^-1^, good anti-inflammatory effect), €: F9-Blank (10 µL.embryo^-1^, uncertain anti-inflammatory effect), (**F**): F9-Blank (20 µL.embryo^-1^, uncertain anti-inflammatory effect), (**G**): Hydrocortisone (20 µg.pellet^-1^, moderate anti-inflammatory effect), (**H**): SDS (20 µg.pellet^-1^, Inactive), (I): Agarose (2.5%, w/v, Inactive).

HP has shown questionable action at the concentration of 10 µg.pellet^-1^, whereas weak activity at 20 µg.pellet^-1^, according to stereomicroscopic evaluations and anti-inflammatory inhibition values (Fig. **7** and Table **9**). When compared to hydrocortisone, a well-known powerful anti-inflammatory agent with a concentration of 20 µg.pellet^-1^ (70.55 0.75%), the F9-HP coded gel formulation (which includes HP methanolic extract) showed a weak anti-inflammatory effect with the inhibition of (58.33 0.29%) at 10 µL.embryo^-1^ and good activity at 20 µL.embryo^-1^ with the inhibition of (76.56). The blank gel exhibited uncertain activity at both concentrations of 10 and 20 µL.embryo-1. The gel exhibited concentration-dependent *in vivo* anti-inflammatory activity, as discussed in the *in vivo* anti-inflammatory results. In the gel formulation, chitosan, previously shown to have anti-inflammatory properties, and HP synergistically exhibit *in vivo* anti-inflammatory potential [96].

## CONCLUSION

Chitosan-based gels containing *Helichrysum pamphylicum* P.H. Davis & Kupicha methanolic extract (HP) were produced successfully. F9-HP, the formulation with the best viscosity and flow properties, was preferred as optimum since the pH value was considered, and the remaining studies were carried out on F9-HP. It was determined that the F9-HP-coded gel was a pseudoplastic flow system. It has been shown that the most suitable model for the F9-HP coded gel formulation is the Power-Law model. This shear-thinning behavior is desirable for topical preparations as they must be thin at the time of application and otherwise thick/solid. The amount of HP was found to be 98.83% ± 0.19 in the F9-HP coded chitosan-based gel), which was thought to prove that the F9-HP coded formulation had a high HP load. The HP release from the F9-HP coded formula was determined to be slower and 9 hours prolonged release of the F9-HP coded formulation compared to pure HP. In line with the release kinetics studies, it was found that the drug delivery system prepared in this study was released by an anomalous (non-fickian) diffusion mechanism. The F9-HP coded formulation significantly showed DPPH free radical scavenger, ABTS•+ cation decolorizing and metal chelating antioxidant activity while weakly reducing antioxidant potential. According to the HET-CAM results, strong anti-inflammatory activity was obtained in the F9-HP coded gel at a dose of 20 µg.embryo^-1^, which increased depending on the dose. It was determined that HP methanol extract and chitosan, at a dose of 20 µg.embryo-1 showed a strong anti-inflammatory effect with a synergistic effect. In conclusion, it can be said that chitosan-based gels containing Helichrysum pamphylicum methanol extract, which can be used in both antioxidant and anti-inflammatory treatment, were successfully formulated and characterized. Different *in vivo* animal and human experiments are planned in the further stages of the study.

## Figures and Tables

**Fig. (1):**
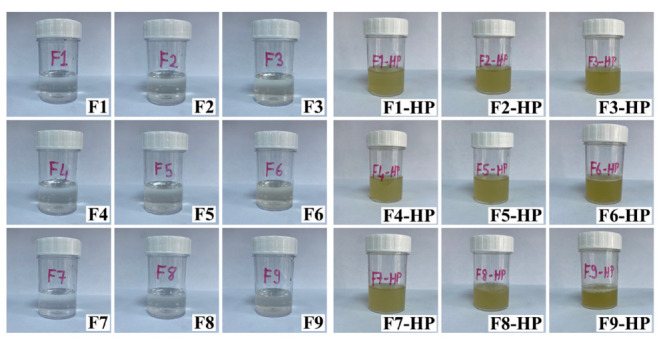
Images of prepared blank formulations and formulations containing HP.

**Fig. (6):**
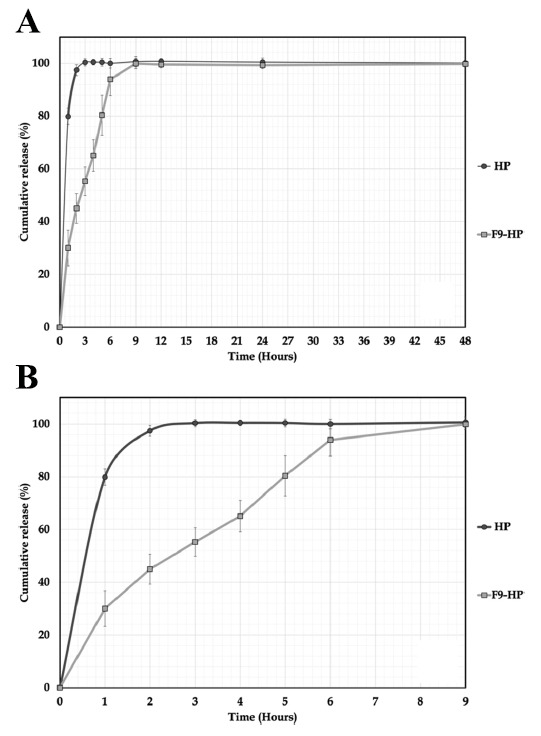
*In vitro* dissolution/release profile of HP and F9-HP. (**A**): 48 h profile, (**B**): 9 h profile.

**Table 1:** Formulation ingredients.

**Code**	**LMW**	**MMW**	**HMW**	**SS**	**SB**	**HP**	**LA**
F1	0.05 g	-	-	0.02 g	0.035 g	10 mg	q.s.
F2	0.10 g	-	-	0.02 g	0.035 g	10 mg	q.s.
F3	0.15 g	-	-	0.02 g	0.035 g	10 mg	q.s.
F4	-	0.05 g	-	0.02 g	0.035 g	10 mg	q.s.
F5	-	0.10 g	-	0.02 g	0.035 g	10 mg	q.s.
F6	-	0.15 g	-	0.02 g	0.035 g	10 mg	q.s.
F7	-	-	0.05 g	0.02 g	0.035 g	10 mg	q.s.
F8	-	-	0.10 g	0.02 g	0.035 g	10 mg	q.s.
F9	-	-	0.15 g	0.02 g	0.035 g	10 mg	q.s.
F1-HP	0.05 g	-	-	0.02 g	0.035 g	10 mg	q.s.
F2-HP	0.10 g	-	-	0.02 g	0.035 g	10 mg	q.s.
F3-HP	0.15 g	-	-	0.02 g	0.035 g	10 mg	q.s.
F4-HP	-	0.05 g	-	0.02 g	0.035 g	10 mg	q.s.
F5-HP	-	0.10 g	-	0.02 g	0.035 g	10 mg	q.s.
F6-HP	-	0.15 g	-	0.02 g	0.035 g	10 mg	q.s.
F7-HP	-	-	0.05 g	0.02 g	0.035 g	10 mg	q.s.
F8-HP	-	-	0.10 g	0.02 g	0.035 g	10 mg	q.s.
F9-HP	-	-	0.15 g	0.02 g	0.035 g	10 mg	q.s.

**Abbreviations:** LMW: Low molecular weight chitosan, MMW: Medium molecular weight chitosan, HMW: High molecular weight chitosan, SS: Sodium salicylate, SB: Sodium bicarbonate, HP: *Helichrysum pamphylicum* P.H.Davis & Kupicha methanolic extract, LA: 1% (w/v) lactic acid aqueous solution, q.s.: sufficient amount.

**Table 2:** The semi-quantitative score system of anti-inflammatory effect on CAM after administration of samples.

**Category**	**Type**	**Effects Observed on CAM after Treatment**
1	Irritated	The granuloma is strongly vascularized. A network of capillaries is formed starlike around the granuloma.
2	Weakly irritated	The granuloma is poorly vascularized. A thin network of capillaries is formed starlike around the granuloma.
3	Weakly normalized	The granuloma is smaller than in categories 1 and 2 and is only poorly vascularized. The starlike network of vessels is hardly recognizable.
4	Normalized	No granuloma or only a kind of “scar” can be observed (if the granuloma regresses, a non-vascularized scar is left). The network of vessels is normal (as the control).

**Table 3:** The proportionate inhibition of inflammation score index.

**İnhibition (%)**	**Anti-inflammatory Effect**
≤ 40	No anti-inflammatory effect
40-55	Uncertain anti-inflammatory effect
55-70	Weak anti-inflammatory effect
70-85	Good anti-inflammatory effect
> 85	Strong anti-inflammatory effect

**Table 4:** Release kinetic modeling and results of F9-HP.

**Model**	**R^2^**	**R^2^_adjusted_**	**MSC**	**AIC**
Korsmeyer-Peppas	0.991	0.990	3.565	31.512
Peppas-Sahlin 1	0.995	0.992	3.803	29.850

**Table 5:** *In vitro* metal chelating activity of samples.

**Code**	**IC_50_ (mg.mL^-1^)**
HP	6.2972
F9-HP	0.3790
F9 (Blank)	0.4180
EDTA	0.1250

**Note:** *nd: no activity detected.

**Table 6:** *In vitro* antioxidant-ABTS radical decolorization activity of samples.

**Code**	**IC_50_ (mg.mL^-1^)**
HP	3.7537
F9-HP	0.5120
F9 (Blank)	1.7060
Ascorbic acid	0.2543
BHT	0.4326

**Note:** *nd: no activity detected.

**Table 7:** *In vitro* reducing powers of samples.

**Code**	**EC_50_ (mg.mL^-1^)**
HP	1.8587
F9-HP	4.8076
F9 (Blank)	nd
Ascorbic acid	0.085
BHT	0.1564

**Note:** *nd: no activity detected.

**Table 8:** *In vitro* antioxidant-DPPH radical scavenging activity of samples.

**Code**	**IC_50_ (mg.mL^-1^)**
HP	0.0023
F9-HP	0.1109
F9 (Blank)	nd
BHT	0.0011

**Note:** *nd: no activity detected.

**Table 9:** The *in vivo* anti-inflammatory effects of samples on the CAM.

**Test Compound**	**Concentration (*µ*g.pellet^-1^) for ** **Standards and (µL.embryo^-1^) for ** **Formulations**	**Average Inhibition^a^ (%)**	**Anti-inflammatory Effect**
HP	10	54.17 ± 0.29	Uncertain
HP	20	62.60 ± 0.83	Weak
F9-HP	10	58.33 ± 0.29	Weak
F9-HP	20	76.56 ± 12.88	Good
F9-Blank	10	43.50 ± 0.68	Uncertain
F9-Blank	20	45.00 ± 0.73	Uncertain
Hydrocortisone	20	70.55 ± 0.75	Moderate
SDS	20	14.27± 0.58	Inactive
Agarose (Blank)	2.5%, w/v	-	Inactive

**Note:**^a^ Values are mean of average score ± Standard deviation (n = 15 for the test substances and n = 10 for standards). *p<0.05 compared with SDS.

## Data Availability

The data and supportive information are available within the article.

## LIST OF ABBREVIATIONS

ABTS: 2,2′-azino-bis (3-ethylbenzothiazoline-6-sulfonic acid)
AIC: Akaike Information Criterion
BHT: Butylated hydroxytoluene
CoF: Coefficients of friction
DMSO: Dimethyl Sulfoxide
DPPH: 2,2-Diphenyl-1-picrylhydrazyl
EDTA: Ethylenediaminetetraacetic acid
FeCl_2_: Iron (II) chloride
FeCl_3_: Iron (III) chloride
HET-CAM: Hen's Egg Test on the Chorioallantoic Membrane
HMW: High molecular weight chitosan
HP: *Helichrysum pamphylicum* P.H. Davis & Kupicha methanolic extract
K_2_S_2_O_8_, K_4_ [Fe: Potassium ferricyanide
(CN)_6_] . 3H_2_O
LMW: Low molecular weight chitosan
MeOH: Methanol
MMW: Medium molecular weight chitosan
MSC: Model Selection Criterion
MWCO: Molecular weight cut-off
nm: Nanometer
PBS: Phosphate-buffered saline
pH: Power of hydrogen
q.s.: Sufficient amount
rpm: Revolutions per Minute
RSD: Relative standard deviation
Rsqr_adj or: Adjusted coefficient of determination
R^2^_adjusted_
Rsqr, R^2^, COD: Coefficient of determination
SB: Sodium bicarbonate
SC: Stratum corneum
SD: Standard deviation
SDS: Sodium dodecyl sulfate
SS: Sodium salicylate
TCA: Trichloroacetic acid
UV: Ultraviolet
Vit C: Vitamin C
